# Expectations of healthcare quality: A cross-sectional study of internet users in 12 low- and middle-income countries

**DOI:** 10.1371/journal.pmed.1002879

**Published:** 2019-08-07

**Authors:** Sanam Roder-DeWan, Anna D. Gage, Lisa R. Hirschhorn, Nana A. Y. Twum-Danso, Jerker Liljestrand, Kwanele Asante-Shongwe, Viviana Rodríguez, Talhiya Yahya, Margaret E. Kruk

**Affiliations:** 1 Harvard T. H. Chan School of Public Health, Department of Global Health and Population, Boston, Massachusetts, United States of America; 2 Ifakara Health Institute, Dar es Salaam, Tanzania; 3 UNICEF, Dar es Salaam, Tanzania; 4 Northwestern University, Feinberg School of Medicine, Chicago, Illinois, United States of America; 5 MAZA, Accra, Ghana; 6 Gillings School of Global Public Health, Department of Maternal and Child Health, University of North Carolina at Chapel Hill, Chapel Hill, North Carolina, United States of America; 7 Bill & Melinda Gates Foundation, Seattle, Washington, United States of America; 8 African Organisation for Research and Training in Cancer, Cape Town, South Africa; 9 Instituto de Efectividad Clínica Sanitaria, Buenos Aires, Argentina; 10 Quality Management Unit, Health Quality Assurance Department, Ministry of Health, Dar es Salaam, Tanzania; University of Cape Town, SOUTH AFRICA

## Abstract

**Background:**

High satisfaction with healthcare is common in low- and middle-income countries (LMICs), despite widespread quality deficits. This may be due to low expectations because people lack knowledge about what constitutes good quality or are resigned about the quality of available services.

**Methods and findings:**

We fielded an internet survey in Argentina, China, Ghana, India, Indonesia, Kenya, Lebanon, Mexico, Morocco, Nigeria, Senegal, and South Africa in 2017 (*N* = 17,996). It included vignettes describing poor-quality services—inadequate technical or interpersonal care—for 2 conditions. After applying population weights, most of our respondents lived in urban areas (59%), had finished primary school (55%), and were under the age of 50 (75%). Just over half were men (51%), and the vast majority reported that they were in good health (73%). Over half (53%) of our study population rated the quality of vignettes describing poor-quality services as good or better. We used multilevel logistic regression and found that good ratings were associated with less education (no formal schooling versus university education; adjusted odds ratio [AOR] 2.22, 95% CI 1.90–2.59, *P* < 0.001), better self-reported health (excellent versus poor health; AOR 5.19, 95% CI 4.33–6.21, *P* < 0.001), history of discrimination in healthcare (AOR 1.47, 95% CI 1.36–1.57, *P* < 0.001), and male gender (AOR 1.32, 95% CI 1.23–1.41, *P* < 0.001). The survey did not reach nonusers of the internet thus only representing the internet-using population.

**Conclusions:**

Majorities of the internet-using public in 12 LMICs have low expectations of healthcare quality as evidenced by high ratings given to poor-quality care. Low expectations of health services likely dampen demand for quality, reduce pressure on systems to deliver quality care, and inflate satisfaction ratings. Policies and interventions to raise people’s expectations of the quality of healthcare they receive should be considered in health system quality reforms.

## Abstract 2

Sanam Roder-DeWan reveal low levels of quality care in some LMICs based on findings of a survey.

## Author summary

**Why was this study done?:**

- Expectations of healthcare quality are believed to influence how patients experience and rate healthcare; however, little is known about expectations in low- and middle-income countries (LMICs).
- High satisfaction with poor-quality care is common in LMICs; one possible explanation is that expectations of care are low.
- This study was conducted to better understand expectations of healthcare quality in LMICs.

**What did the researchers do and find?:**

- We used an innovative internet sampling methodology to collect information from 17,996 individuals in 12 LMICs.
- Vignettes describing poor-quality care were used to elicit expectations of healthcare; good ratings for poor-quality care approximate low expectations.
- Over half of respondents (53%) rated the poor-quality care vignettes as good quality or better; low expectations were more likely if respondents were less educated, were male, reported good personal health, or had experienced discrimination during a healthcare visit in the past.

**What do these findings mean?:**

- Internet users in LMICs are a relatively privileged subgroup in our study countries, limiting the generalizability of our findings.
- Populations with low expectations are more likely to be satisfied with poor-quality care, reducing the demand-side pressure that health systems experience to deliver high-quality care.
- Raising expectations of quality may be one approach to improving the quality of healthcare in LMICs.

## Introduction

A growing body of literature describes systematically poor quality of healthcare in low- and middle-income countries (LMICs) today [1–4]. For example, only 21% of providers correctly managed tuberculosis in a study using standardized patients in India [5]. Health workers in 18 LMICs performed on average less than half of recommended reproductive, maternal, newborn, and child health actions during a visit, and a patient in Africa is twice as likely to die after surgery than the global average [2,6]. An analysis of global data estimated that 8.6 million lives lost in LMICs in 2016 could have been prevented by high-quality healthcare; whereas 40% did not have access to care, 60% made it to a facility but did not receive the high-quality care needed to avert death [7]. Nonhealth outcomes such as confidence in the health system and cost of care also suffer in settings of low quality [8].

Despite this, satisfaction with care has been generally high [2,9]. People’s satisfaction with care is related to their expectations of quality, and these expectations can be lowered by information asymmetry (i.e., not knowing what elements of care delivery are optimal) or lack of experience with high-quality services in their environment [10–12]. Low agency and disempowerment may further depress expectations: a range of studies have found that poor and less educated respondents are more likely to rate care as satisfactory [9,11,13,14].

Low expectations are problematic for several reasons. One, if people expect poor-quality care, either because they do not know what high-quality care is or because they have become accustomed to poor-quality care, they are less likely to hold health systems accountable for poor performance. This is a missed opportunity to improve healthcare through feedback. In addition, people with low expectations are less effective in seeking better care. A growing literature in health economics and health services research has found that “active” patients, those who make strategic decisions about where to access care in an effort to receive higher quality services, are able to extract higher quality care from the system [15,16]. They select, bypass, and abandon care based on whether or not a facility is perceived to be able to meet their expectations of quality [15,17,18]. Thus, raising expectations may result in more people obtaining better care and provide feedback to health systems for improvement. Finally, measures of health quality expectations can be used as anchoring vignettes to permit better comparison of self-reported service quality and satisfaction across countries [19–21].

Despite the importance of understanding expectations of healthcare quality, this concept is undertheorized and has been little researched in LMICs [9,11,22]. To address this gap, we assessed the ratings of quality for standardized healthcare vignettes designed to portray poor-quality care among internet users in LMICs. These ratings are considered a measure of expectations of quality. We explored associations between good-quality ratings and user and healthcare factors.

## Methods

### Study design

We fielded an internet survey to explore healthcare expectations in 12 LMICs: Argentina, China, Ghana, India, Indonesia, Kenya, Lebanon, Mexico, Morocco, Nigeria, Senegal, and South Africa (see S1 Appendix and S2 Appendix for survey instrument). The study countries were selected because they represent a variety of world regions and comprise a large share of populations living in LMICs. All study countries had internet penetrance over 20% (S3 Appendix). Our original analysis plan for this data set included the research question pertinent to the current analysis, “What are expectations of healthcare in LMICs across socio-demographic and contextual factors?” (S4 Appendix).

We asked web users aged 18 or older about demographics, healthcare utilization, perceptions of healthcare quality, and healthcare vignettes describing poor quality of care. Respondent location was determined using internet protocol (IP) addresses. We collected data during August and September of 2017. Translators translated the survey into local languages, and native speakers then back-translated into English to check for accuracy (S5 Appendix).

Internet surveys allow for collection of a large number of responses across countries while minimizing social desirability bias [23]. We used Random Domain Intercept Technology (RDIT) to reach a wide population of internet users. RDIT “intercepts” internet users who have entered the name of a site that does not exist or has expired and invites them to complete the survey. RDIT produces a sample that is highly representative of the internet-using population [24]. The method has been found to produce stable findings over time. For example, a mental health survey repeatedly conducted in India every month for over 21 months produced consistent estimates with low standard errors as did a vaccine belief survey in Ontario [25–27].

We used several strategies to ensure validity of the responses. IP addresses were monitored to avoid duplicate responses, and proprietary code prevented automated entries by “bots.” In order to hold the respondent’s attention and to reduce thoughtless clicking, we kept the survey short, randomly varied the order of categorical responses, and moved the location of the questions and responses on the screen (see S6 Appendix for a further discussion of our internet survey methodology).

**Fig 1:**
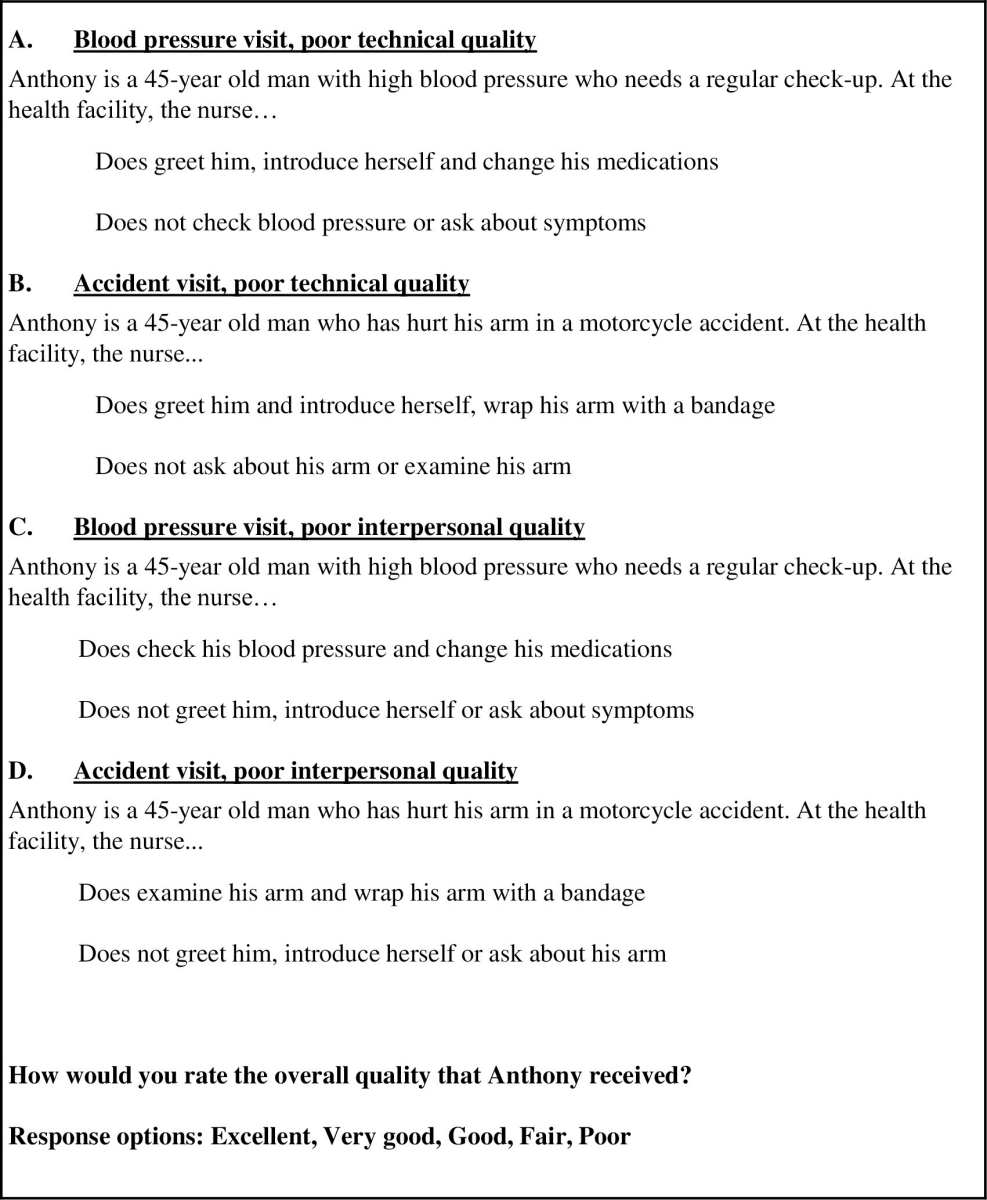
Vignettes describing poor-quality care. All respondents received vignette A. A subset of respondents also received vignette B, C, or D. Please see S1 Appendix for full survey instrument and S2 Appendix for survey screenshot.

The main study outcome is the respondent’s rating of vignettes illustrating poor quality of healthcare in the domains of technical quality (competence) or interpersonal quality (communication, respectful treatment). A good, very good, or excellent rating is considered a measure of low expectation of service quality. All respondents were shown a vignette about a routine clinic visit for hypertension management (Fig 1). In this visit, the nurse does not check the patient’s blood pressure or ask about his symptoms but changes his medicine and is courteous (“A. blood pressure visit; poor technical quality”). Respondents also received 1 of 3 additional vignettes. The second vignette also describes poor technical quality of care; a patient is seen for an arm injury caused by an accident. The patient’s arm is not examined, and he is not asked about his symptoms (“B. accident visit; poor technical quality”). This vignette was included to explore the effect of the specific health condition on quality ratings. The last 2 vignettes use the same clinical conditions as above but describe poor interpersonal quality of care (“C. blood pressure visit; poor interpersonal quality” and “D. accident visit; poor interpersonal quality”). The last 2 vignettes were included to test the impact of interpersonal quality deficits on overall ratings.

The 4 vignettes were intentionally designed to describe poor quality of care that is discernable to the respondent. Although lay people are unlikely to be able to judge provider adherence to clinical guidelines or choice of correct treatment, the literature has shown that they understand the importance of clinician assessment, including thoroughness of history and physical exam [28–30]. To further assist the respondents in identifying the nature of clinical assessment that is indicated in a visit for hypertension or injury, we identified items that were not completed in the visit. Two of our 4 vignettes describe poor interpersonal quality of care (C. and D.); lay people are expert judges of this, and surveys of patient perceptions of quality, such as the Consumer Assessment of Healthcare Providers and Systems, routinely ask about interpersonal quality of care. Respondents were asked to rate the overall quality of care described in the vignettes using a 5-point categorical response scale (excellent, very good, good, fair, and poor). We defined low expectations as a rating of excellent, very good, or good.

Individual and health system variables believed to influence expectations of healthcare were selected based on the literature on the determination of satisfaction and quality ratings (S7 Appendix). These include demographics, health, nature of local health system, past care experience, and attitudinal positivity [9,11,14,31,32]. We included gender, age, urban or rural residence, and educational attainment. Self-reported general and mental health status were rated using a 5-point categorical response scale (excellent, very good, good, fair, and poor). Educational attainment response options were as follows: completed college or university, some college or university, secondary or high school completed, some secondary or high school, primary school completed, some primary school, or no formal schooling.

Experience with the health system included frequency of use (number of outpatient visits over the last year) and past treatment (having “ever been discriminated against, hassled, or made to feel inferior by a health provider/staff”). Although it is hypothesized that information about healthcare and healthcare quality is likely to influence expectations, our survey did not allow us to explore this factor.

### Analysis

We calculated descriptive statistics for survey respondent characteristics across and within countries for the variables of interest. Weights using age, gender, urban or rural residence, and educational attainment were created to approximate national populations (see S8 Appendix and S9 Appendix). We tested the association between the predictors and good or better quality ratings using multilevel random intercept logistic regression. We include an ordinal logistic model in the appendix for reference (S10 Appendix), although this model was rejected because the assumption of proportional odds was violated. The psychological literature shows that individual personality and tendency towards positivity or negativity affect both survey reporting behavior and expectations [14,33]. Previous studies of patient health preferences have used mental health status to approximate positivity or negativity [14]. Given this literature, we performed an additional analysis in which we added self-reported mental health status to our model. Though self-reported mental health is not a direct proxy for positivity or negativity, personality is likely to play some role in reporting mental health status. To explore potential patterns in the influence of country income or health system performance on expectations, we conducted several supplementary analyses in which we regressed ratings of quality on country income and the Healthcare Access and Quality (HAQ) index of health system performance [34]. All regressions used unweighted data. Data analysis was completed in Stata/SE version 14.2 (StataCorp, College Station, Texas). This study (protocol number IRB17-0907) was reviewed and determined to be exempt by the Harvard University Human Research Protection Program.

## Results

Of the 57,786 respondents who opted to take the survey, 17,996 respondents (31%) completed the survey questions for this analysis; this formed our analytic sample (Table 1). The completion rate is similar to rates reported for similar studies [35–38]. After weighting the data with population census weights, approximately half of respondents (51%) were male, 35% were between the ages of 18 and 29, 39% were between the ages of 30 and 49, and 25% were over the age of 50. Rural residents made up 41% of the sample. Nearly half of respondents (45%) had a primary education or less. The vast majority (73%) reported good general health with a mean number of outpatient healthcare visits over the last year of 2.5 (SD 3.0). One-third of all respondents (34%) reported that they experienced discrimination in the healthcare system in the past.

**Table 1:** Sample characteristics with population weights.

Number of respondents	Argentina (*N* = 1,377)	China (*N* = 1,803)	Ghana (*N* = 1,366)	India (*N* = 1,901)	Indonesia (*N* = 1,586)	Kenya (*N* = 1,386)	Lebanon (*N* = 1,369)	Mexico (*N* = 1,520)	Morocco (*N* = 1,455)	Nigeria (*N* = 1,517)	Senegal (*N* = 1,292)	South Africa (*N* = 1,424)	Total (*N* = 17,996)
**Socio-demographics**													
**Male (*N*; %)**	658; 48%	999; 55%	735; 54%	992; 52%	700; 44%	723; 52%	709; 52%	743; 49%	715; 49%	1,018; 67%	553; 43%	681; 48%	9,225; 51%
**Rural (*N*; %)**	91; 7%	726; 40%	599; 44%	1,125; 59%	851; 54%	1,062; 77%	164; 12%	278; 18%	563; 39%	654; 43%	845; 65%	498; 35%	7,457; 41%
**Primary education or less (*N*; %)**	584; 42%	538; 30%	490; 36%	1,076; 57%	758; 48%	898; 65%	539; 39%	550; 36%	891; 61%	483; 32%	1,096; 85%	248; 17%	8,150; 45%
**Good self-reported health status**^**a**^ **(*N*; %)**	1,080; 78%	1,297; 72%	1,109; 81%	1,260; 66%	999; 63%	951; 69%	1,144; 84%	1,176; 77%	855; 59%	1,341; 88%	729; 56%	1,171; 82%	13,114; 73%
**Age: 18–29 (*N*; %)**	314; 23%	480; 27%	611; 45%	713; 38%	493; 31%	601; 43%	411; 30%	466; 31%	432; 30%	810; 53%	505; 39%	518; 36%	6,354; 35%
**Age: 30–49 (*N*; %)**	497; 36%	807; 45%	417; 31%	796; 42%	630; 40%	522; 38%	600; 44%	620; 41%	608; 42%	546; 36%	464; 36%	598; 42%	7,103; 39%
**Age: 50+ (*N*; %)**	566; 41%	517; 29%	338; 25%	391; 21%	463; 29%	264; 19%	358; 26%	434; 29%	415; 28%	161; 11%	323; 25%	308; 22%	4,539; 25%
**Experience with healthcare system**													
**Ever experienced discrimination**^**b**^ **(*N*; %)**	301; 22%	680; 38%	427; 31%	632; 33%	526; 33%	469; 34%	411; 30%	430; 28%	613; 42%	678; 45%	549; 43%	415; 29%	6,131; 34%
**Number of visits in past year**^**c**^ **(mean; SD)**	3.0; 3.2	1.9; 2.7	2.0; 2.5	3.4; 3.6	2.2; 2.9	2.5; 2.9	1.8; 2.6	2.8; 3.0	2.4; 3.2	2.7; 3.0	1.8; 2.8	2.9; 3.2	2.5; 3.0

Please see Fig 1 for vignette language. For reference, please see S8 Appendix for country demographics.

^a^Good self-reported general health status is defined as a response of good, very good, or excellent to the question “In general, would you say your health is…”

^b^The question regarding discrimination was “Have you ever been discriminated against, hassled, or made to feel inferior by a health provider/staff for any of these reasons?”

^c^The prompt for number of visits was “In the past year, how many times did you go to a clinic, health center, or hospital to receive health care for yourself? (Please do not include any times you stayed overnight.).”

**Fig 2:**
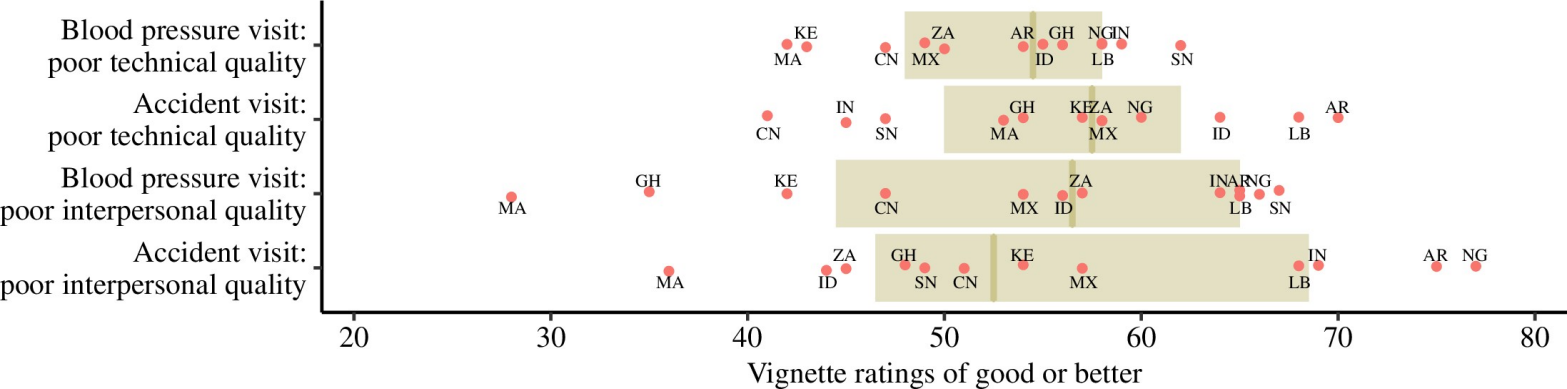
Ratings of good or better on vignettes describing poor-quality care. Blood pressure visit, poor technical quality vignette: *N* = 17,996. Accident visit, poor technical quality vignette: *N* = 3,640. Blood pressure visit, poor interpersonal quality vignette: *N* = 3,541. Accident visit, poor interpersonal quality vignette: *N* = 3,667. AR, Argentina; CN, China; GH, Ghana; ID, Indonesia; IN, India; KE, Kenya; LB, Lebanon; MA, Morocco; MX, Mexico; NG, Nigeria; SN, Senegal; ZA, South Africa.

Over half (53%) of respondents across countries rated the quality of care described in vignette A (blood pressure visit; poor technical quality) as good or better (Fig 2). This rate was similar when varying the health condition; 55% rated vignette B (accident visit; poor technical quality) as good or better. In the variations of the 2 vignettes in which interpersonal quality is poor, 54% (C. high blood pressure; poor interpersonal quality) and 57% (D. accident: poor interpersonal quality) gave a rating of good or better. Respondents from Senegal were most likely to rate vignette A (blood pressure visit; poor technical) as good or better.

Men had lower expectations than women in our sample (Table 2). The following variables increased the odds of rating vignette A(blood pressure visit; poor technical quality) as good or better: male gender (adjusted odds ratio [AOR] 1.32, 95% CI 1.23–1.41, *P* < 0.001), no formal schooling (AOR 2.22, 95% CI 1.90–2.59, *P* < 0.001), excellent self-reported health (AOR 5.19, 95% CI 4.33–6.21, *P* < 0.001), and history of discrimination in healthcare (AOR 1.47, 95% CI 1.36–1.57, *P* < 0.001). There were small differences in these predictors across the 4 vignettes. Results of the multilevel models confirmed significant differences across countries. Supplementary analyses exploring effects of country income or health system performance on expectations did not suggest a clear pattern. We believe that heterogeneity of preferences across populations is influenced by a large range of factors, including prevailing models of care, utilization patterns, media, and political factors. The supplementary analysis, which included self-reported mental health in our multilevel model for vignette A, found that those with excellent self-reported mental health had nearly 4 times higher odds of having low expectations of quality (S11 Appendix).

**Table 2:** Determinants of good ratings of poor quality.

	Blood pressure visit; poor technical quality (*n* = 17,996)			Accident visit; poor technical quality (*n* = 3,640)			Blood pressure visit; poor interpersonal quality (*n* = 3,541)			Accident visit; poor interpersonal quality (*n* = 3,667)		
	AOR	95% CI	*P* value	AOR	95% CI	*P* value	AOR	95% CI	*P* value	AOR	95% CI	*P* value
**Individual characteristics**												
Male gender	1.32	1.23–1.41	<0.001	1.28	1.09–1.49	< .01	1.46	1.25–1.72	< .001	1.21	1.04–1.42	< .001
Age	1.01	1.00–1.01	<0.001	1.01	1.00–1.01	.03	1.01	1.00–1.01	0.077	1.00	1.00–1.01	0.216
**Educational attainment (reference: completed college or university)**												
Some college or university	1.19	1.09–1.30	<0.001	1.13	0.93–1.37	0.23	1.18	0.97–1.44	0.10	1.16	0.95–1.42	0.14
Secondary/ high school completed	1.40	1.29–1.53	<0.001	1.51	1.24–1.83	<0.001	1.42	1.17–1.74	<0.001	1.38	1.13–1.67	<0.01
Some secondary or high school	1.82	1.63–2.04	<0.001	1.86	1.45–2.38	<0.001	1.76	1.35–2.28	<0.001	2.02	1.56–2.60	<0.001
Primary school completed	2.13	1.80–2.53	<0.001	2.21	1.51–3.24	<0.001	3.81	2.47–5.89	<0.001	1.74	1.19–2.55	<0.01
Some primary school	2.70	2.16–3.36	<0.001	3.96	2.31–6.77	<0.001	2.01	1.23–3.26	<0.01	1.87	1.11–3.15	0.02
No formal schooling	2.22	1.90–2.59	<0.001	1.73	1.23–2.42	<0.01	2.18	1.51–3.15	<0.001	1.50	1.06–2.12	0.02
Rural residence	0.98	0.90–1.08	0.70	0.99	0.82–1.21	0.95	0.90	0.73–1.11	0.34	0.91	0.73–1.12	0.36
**Self-reported health (reference: poor)**												
Fair	1.05	0.87–1.27	0.61	1.26	0.82–1.94	0.29	1.08	0.71–1.66	0.71	1.09	0.73–1.64	0.68
Good	2.34	1.96–2.79	<0.001	3.07	2.06–4.58	<0.001	2.59	1.74–3.86	<0.001	2.20	1.51–3.20	<0.001
Very good	2.95	2.46–3.54	<0.001	3.25	2.16–4.89	<0.001	3.8	2.53–5.71	<0.001	3.22	2.19–4.75	<0.001
Excellent	5.19	4.33–6.21	<0.001	6.27	4.17–9.42	<0.001	5.61	3.74–8.43	<0.001	6.22	4.21–9.18	<0.001
Number outpatient visits past year^a^ (reference: none)	1.04	1.03–1.05	<0.001	1.06	1.03–1.09	<0.001	1.03	1.01–1.06	0.010	1.06	1.03–1.08	<0.001
Ever experienced discrimination^b^ (reference: no discrimination)	1.47	1.36–1.57	<0.001	1.26	1.08–1.48	0.01	1.35	1.15–1.59	<0.001	1.10	0.93–1.29	0.26
**Country characteristics (12 countries)**												
Country variance	0.09	0.04–0.23		0.05	0.02–0.13		0.20	0.08–0.50		0.19	0.07–0.54	
Likelihood ratio test versus logistic model^c^ (Prob. ≥ chibar2)	344.11		<0.001	26.72		<0.001	122.64		<0.001	121.89		<0.001

These results are from a random intercept multilevel logistic regression. Coefficients are the AOR of having low expectations of care (defined as a rating of good or better on the vignettes). Data are unweighted.

^a^The prompt for number of visits was “In the past year, how many times did you go to a clinic, health center, or hospital to receive health care for yourself? (Please do not include any times you stayed overnight.).”

^b^The question regarding discrimination was “Have you ever been discriminated against, hassled, or made to feel inferior by a health provider/staff for any of these reasons?”

^c^The likelihood ratio test compares this model to an ordinary logistic model and is significant for all outcomes. This supports the decision to use multilevel models.

**Abbreviation:** AOR, adjusted odds ratio

## Discussion

Responses from nearly 18,000 internet users in 12 LMICs show that good ratings for poor quality are common: over 50% of respondents indicated that objectively poor quality of care described in vignettes was good, very good, or excellent. Vignettes highlighting poor technical quality and poor interpersonal quality yielded similar results, supporting the hypothesis that low expectations, not lack of technical knowledge, drives these ratings. The prevalence of good ratings is especially notable given that internet users are likely to be more affluent and educated than the general population and thus more likely to access better quality care and have higher expectations of quality [40,41]. Our finding that a majority of people in the study countries have low expectations of healthcare quality points to a lost opportunity to keep health systems accountable for the quality of care that they deliver. This work may help explain current high satisfaction ratings and inform efforts to better measure people’s assessment of health system performance.

We found significant variation in expectations across countries both in our analytic models and in unadjusted comparisons. Greater differences between countries were noted for the vignette describing poor interpersonal quality, perhaps because of the more subjective and socio-culturally influenced nature of interpersonal quality of care. Health system factors probably play an important role in this variation. For example, of all country respondents, the Senegalese were most likely to rate both hypertension vignettes as good or better; their ratings of both accident vignettes were below the full sample average (Fig 2). Healthcare utilization for injury is higher than that for (diagnosed) hypertension in Senegal [42,43]. Could the Senegalese pattern be due to respondent familiarity with the 2 conditions? High ratings for poor quality were also frequent in India but in only 3 of 4 vignettes without a clear explanation for the pattern—Indian respondents appear to be most sensitive to poor technical quality during a visit for an injury. Relatively low health system investment and documented poor quality of care in both public and private sectors in India would have predicted chronic exposure to poor-quality care and lower expectations across all 4 vignettes [44–46]. Moroccan respondents consistently had some of the lowest ratings for the poor-quality vignettes. This may be driven by remarkable recent improvements in health outcomes in Morocco [47,48]. However, Kenyan respondents also had lower ratings despite less progress on achieving broad-based health gains [49]. Higher expectations in Kenya may have been shaped by recent countrywide healthcare strikes [50]. A complex interplay between political, social, economic, and health system factors is likely to explain these finding, and national differences require further research.

Male gender, low education, experience of discrimination in the health system, and good health status were associated with low expectations of care in adjusted models for all versions of the vignette. Respondents with no primary education were 2 times as likely to have low expectations than their more educated peers. This is consistent with existing literature on expectations and quality ratings [22,51]. Satisfaction with maternity services and quality ratings, for example, are lower in respondents with higher educational levels in various LMIC settings [52–54], whereas expectations of good patient-doctor communication are higher [22,51]. Low education both represents an information deficit and is likely to be linked to low socioeconomic status. Respondents with low social status may have lower expectations because of chronic and repeated exposure to low-quality services as well as less access to accurate information about the health system [2,55]. Low expectations may also stem from a more general experience of disempowerment and lack of entitlement to other government services [56].

We found that women had higher expectations of quality than men. Their higher expectations may be due to frequency and nature of interactions with the health system: women are often the primary caregivers in families and more likely to interact with the healthcare system for maternal and child health services [57,58]. Women may place a higher value on healthcare because they are often responsible for sick children, particularly vulnerable newborns, and personally experience the potentially life-threatening event of childbirth. Though our model included the number of outpatient visits in the past year, we did not have information on the nature of care received, and it is possible that this was systematically different for men and women. Another possible explanation is that women have better access to information because they are frequently targeted for health education. The lack of association with age is consistent with a study of pre- and post-visit expectations in the United Kingdom that showed that older adults did not have lower expectations of care but that they were more likely to believe that their expectations had been met [11]. This last point is supported by relative agreement in the literature on the role of age on patient satisfaction; satisfaction increases with age [31].

**Fig 3:**
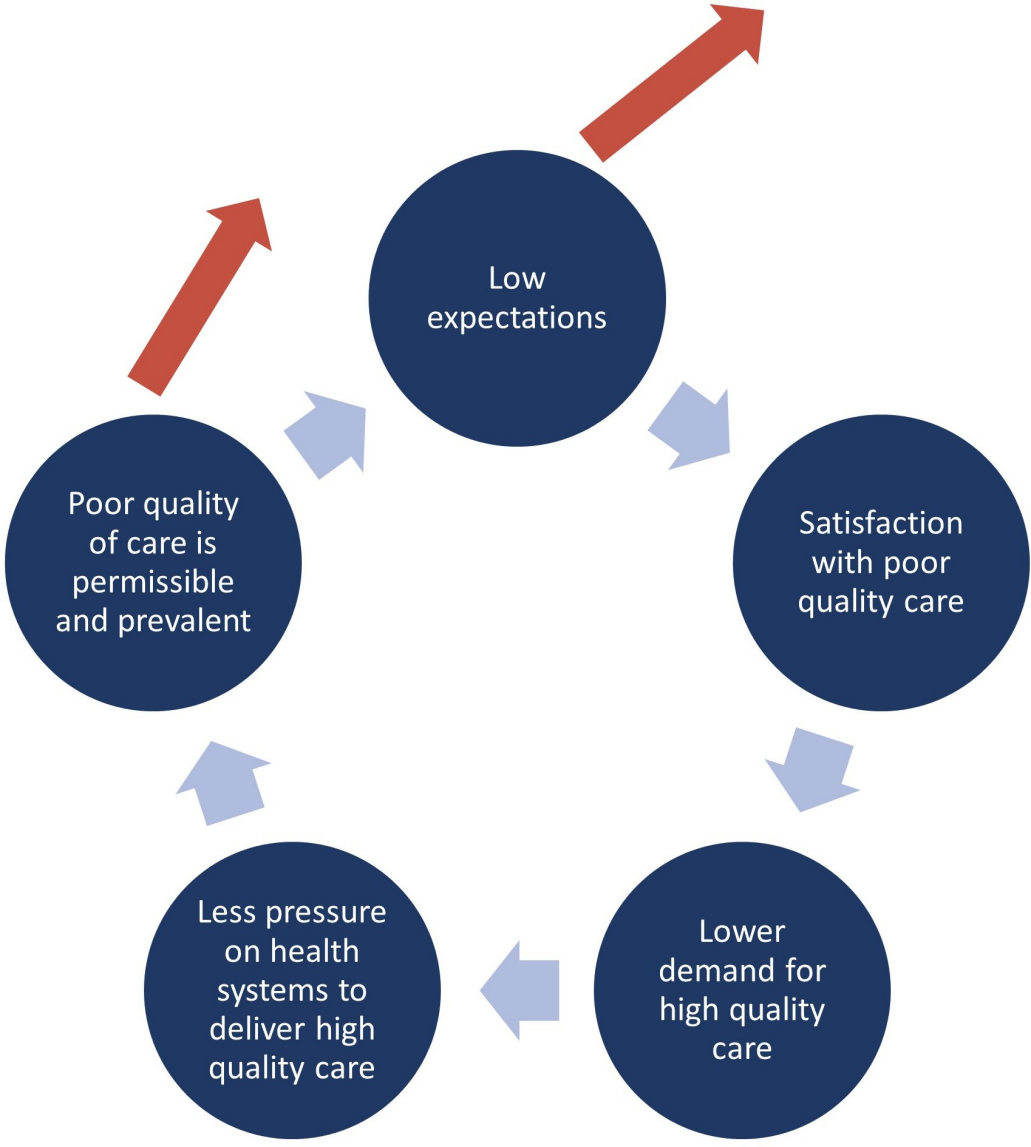
The cycle of low expectations and poor quality of care. This analysis suggests that there may be a vicious cycle of low expectations and poor quality of care. The cycle may be broken by delivering high-quality care or by raising expectations of quality.

Regarding experience with the health system, more outpatient visits in the last year is associated with good ratings for poor-quality care, but the increased odds are small. Without knowing the quality of those visits, it is difficult to interpret this result. However, there is a growing body of evidence that describes substantial deficits in the quality of care in our study countries, making it likely that additional experience with healthcare is experience with poor-quality healthcare [2,59–61]. More directly, our results showed that a history of discrimination raised the likelihood of good ratings for poor quality by nearly 50% for the main vignette, suggesting that a history of poor-quality care is associated with lower expectations. This is consistent with the literature showing that a person’s experience with healthcare is the most important source of information about quality and a strong driver of perceptions of that care [54,62]. It is possible that experience of poor-quality care may stifle the demand for high-quality care and create a vicious cycle of low expectations and poor-quality care (Fig 3). Conversely, improvements in quality of care or raising people’s expectations may break this cycle, possibilities that require further study.

Better self-reported health status was strongly associated with low expectations of care. This is consistent with the hypothesis that health status would be inversely related to expectations of care. Healthier people need less from the health system and may have a more positive outlook. In contrast, sicker patients may place a higher value on healthcare, making them less tolerant of poor quality. Research from a variety of different countries has shown that patients who actively navigate the health system are more likely to have a serious health condition [15,16,62]. In our subanalysis including self-reported mental health status, the variable was strongly associated with low expectations, suggesting that the role of individual outlook (overall positivity or negativity) in shaping expectations warrants further assessment.

Further research is needed to fully understand how expectations of quality can be increased and whether these higher expectations can contribute to health system quality improvement in LMICs. Studies of expectations focusing on equity and specifically targeting marginalized groups will be especially important for understanding the role of disempowerment on expectations of care.

The report of the Lancet Global Health Commission on High Quality Health Systems recommends that governments consider “igniting demand” for high-quality care as 1 of 4 universal actions to improve quality at scale [2]. Understanding people’s expectations of quality and how to raise these expectations will be important in raising demand for quality. We were unable to explore the role that information about the health system—i.e., educating people about their entitlements or information about what good care consists of—plays in shaping expectations. From an improvement perspective, information about the health system is potentially the most malleable of the factors influencing expectations. It has also been shown to drive decisions about care seeking [63]. Information as a lever for raising expectations of quality may be especially important in poorly functioning health systems, in which experience with poor quality is likely to be prevalent and to lower expectations.

Evidence for specific interventions that raise healthcare expectations is still sparse [2]. Several intervention types show the potential to raise expectations and suggest that higher expectations through information sharing are involved in improving quality. For example, participatory women’s groups are associated with better provider practices during childbirth, partially because women are learning to expect proper hygienic care and demanding it [64]. Consumer quality reporting, patients-rights charters, and mass media campaigns are also promising approaches that warrant further exploration. Increasing quality and transparency of information is an especially timely area for further research and potential action because of the recent growth in mechanisms for information sharing and patient involvement created by the digital revolution. People are increasingly using the internet and mobile phones to share and receive information on health and healthcare quality [65,66]. Program planners are also leveraging the widespread use of mobile technology to engage people [67].

This study has several limitations. Internet surveys in countries with generally low internet penetration are not representative of the full population because internet users are likely to be male, wealthy, young, and more urban than the general population [36]. Based on our conceptual framework, we believe that our sample is likely to have higher expectations than the general population in our survey countries. Population weights have been applied to our descriptive statistics, but this cannot compensate for the absence of entire demographic groups, and thus our inference is limited to the internet-using population. Internet surveys are also known to have lower response rates than face-to-face surveys, leading to concerns of nonresponse bias [37]. We addressed this by surveying a large sample, limiting the length of the questions, and structuring the survey for ease of response. On the other hand, internet surveys are useful for exploring sensitive topics due to low social desirability and acquiescence biases, which may lead to more honest responses about healthcare [39]. We were unable to directly assess respondent wealth, which is likely to play a role in expectations of quality that may be distinct from education. Additionally, social, community, and healthcare context was only approximated in our model by country, and we were unable to assess the respondents’ access to information about quality healthcare. Finally, concerns about cognitive overload for the respondent meant that we elected not to include a “control” vignette describing only high-quality care.

Our findings suggest that expectations of quality are low and that caution is needed when interpreting satisfaction and other health system ratings because they are likely to be biased upward. Vignettes such as ours that establish people’s expectations, values, and preferences for healthcare can be used as anchoring vignettes to rescale satisfaction ratings. Anchoring vignettes allow researchers to control for people’s internal standards of quality and make more accurate comparisons and interpretations of satisfaction and quality ratings [20,21]. These vignettes can also help policy makers accurately gauge the impact of new policies and interventions on the quality of care in their health systems.

To our knowledge, this is the first multicountry study of expectations of healthcare quality in LMICs. Our results show that people’s ratings of poor quality are remarkably high in LMIC settings, with over 50% of respondents rating vignettes describing poor-quality care as good or better. This points to an opportunity for future efforts to improve health systems. Increasing expectations of good care from the public should exert much needed pressure on health systems to provide competent and respectful care. Raising expectations should be part of the broader health system improvement agenda as countries adopt universal health coverage.

## Supporting information

S1 Appendix Survey instrument. (DOCX)

S2 Appendix Survey instrument screen shot. (DOCX)

S3 Appendix Internet penetration rates in survey countries. (DOCX)

S4 Appendix Original analysis plan. (DOCX)

S5 Appendix Survey language. (DOCX)

S6 Appendix Expanded rationale for methods. (DOCX)

S7 Appendix Conceptual framework for expectations of quality. (DOCX)

S8 Appendix Country demographic data. (DOCX)

S9 Appendix Weights. (DOCX)

S10 Appendix Ordered logistic regression. (DOCX)

S11 Appendix Determinants of good ratings for poor-quality subanalysis, including self-reported mental health status. (DOCX)

S12 Appendix STROBE checklist. STROBE.
(DOCX)

## Abbreviations

AOR: adjusted odds ratio
HAQ: Healthcare Access and Quality
IP: internet protocol
LMIC: low- and middle-income country
RDIT: Random Domain Intercept Technology

## References

[1] Kruk ME, Leslie HH, Verguet S, Mbaruku GM, Adanu RMK, Langer A. Quality of basic maternal care functions in health facilities of five African countries: an analysis of national health system surveys. The Lancet Global Health. 2016;4(11):e845–e55. 10.1016/S2214-109X(16)30180-2 27670090

[2] Kruk ME, Gage AD, Arsenault C, Jordan K, Leslie HH, Roder-Dewan S, et al High-quality health systems in the Sustainable Development Goals era: time for a revolution. The Lancet Global Health. 2018;6(11):e1196–e252. 10.1016/S2214-109X(18)30386-3 30196093 PMC7734391

[3] Berendes S, Heywood P, Oliver S, Garner P. Quality of Private and Public Ambulatory Health Care in Low and Middle Income Countries: Systematic Review of Comparative Studies (Quality of Private Versus Public Care). PLoS Med. 2011;8(4):e1000433 10.1371/journal.pmed.1000433 21532746 PMC3075233

[4] World Health Organization; World Bank Group; OECD. Delivering Quality Health Services: A Global Imperative for Universal Health Coverage: Geneva: World Health Organization; 2018.

[5] Das J, Kwan A, Daniels B, Satyanarayana S, Subbaraman R, Bergkvist S, et al Use of standardised patients to assess quality of tuberculosis care: a pilot, cross-sectional study. Lancet Infect Dis. 2015;15(11):1305–13. Epub 2015/08/14. 10.1016/S1473-3099(15)00077-8 26268690 PMC4633317

[6] Biccard BM, Madiba TE, Kluyts H-L, Munlemvo DM, Madzimbamuto FD, Basenero A, et al Perioperative patient outcomes in the African Surgical Outcomes Study: a 7-day prospective observational cohort study. The Lancet. 2018;391(10130):1589–98. 10.1016/S0140-6736(18)30001-1 29306587

[7] Kruk ME, Gage AD, Joseph NT, Danaei G, García-Saisó S, Salomon JA. Mortality due to low-quality health systems in the universal health coverage era: a systematic analysis of amenable deaths in 137 countries. Lancet (London, England). 2018;392(10160):2203 10.1016/S0140-6736(18)31668-4 30195398 PMC6238021

[8] Rockers PC, Kruk ME, Laugesen MJ. Perceptions of the health system and public trust in government in low- and middle-income countries: evidence from the World Health Surveys. Journal of health politics, policy and law. 2012;37(3):405–37. Epub 2012/02/11. 10.1215/03616878-1573076 . 22323234

[9] Batbaatar E, Dorjdagva J, Luvsannyam A, Amenta P. Conceptualisation of patient satisfaction: a systematic narrative literature review. Perspectives in Public Health. 2015;135(5):243–50. 10.1177/1757913915594196 26187638

[10] Murray C, Kawabata K, Valentine N. People's experience versus people's expectations. Health Affairs. 2001;20(3):21–4. 10.1377/hlthaff.20.3.21 11585169

[11] Bowling A, Rowe G, McKee M. Patients’ experiences of their healthcare in relation to their expectations and satisfaction: a population survey. Journal of the Royal Society of Medicine. 2013;106(4):143–9. 10.1258/jrsm.2012.120147 23564898 PMC3618164

[12] Conway T, Willcocks S. The role of expectations in the perception of health care quality: developing a conceptual model. International journal of health care quality assurance 1997;10(2–3):131. 10.1108/09526869710167058 10169233

[13] Bowling A, Rowe G, Lambert N, Waddington M, Mahtani KR, Kenten C, et al The measurement of patients' expectations for health care: a review and psychometric testing of a measure of patients' expectations. Health technology assessment (Winchester, England). 2012;16(30):i 10.3310/hta16300 22747798

[14] Bleich SN, Ozaltin E, Murray CJL. How does satisfaction with the health-care system relate to patient experience?/Quel lien existe-t-il entre la satisfaction a l'egard du systeme de sante et l'experience des soins vecue par les patients?/Relacion entre la satisfaccion con el sistema de atencion sanitaria y la experiencia personal de los pacientes.(Research). Bulletin of the World Health Organization. 2009;87(4):271 10.2471/BLT.07.050401 19551235 PMC2672587

[15] Leonard KL. Active patients in rural African health care: implications for research and policy. Health Policy and Planning. 2014;29(1):85–95. 10.1093/heapol/czs137 23307907

[16] Cohen J, Golub G, Kruk ME, McConnell M. Do active patients seek higher quality prenatal care?: A panel data analysis from Nairobi, Kenya. Preventive Medicine. 2016;92:74–81. 10.1016/j.ypmed.2016.09.014 27667338 PMC5100690

[17] Kruk ME, Hermosilla S, Larson E, Mbaruku GM. Bypassing primary care clinics for childbirth: a cross-sectional study in the Pwani region, United Republic of Tanzania. Bull World Health Organ. 2014;92(4):246–53. 10.2471/BLT.13.126417 24700992 PMC3967574

[18] Roder-DeWan S, Gupta N, Kagabo DM, Habumugisha L, Nahimana E, Mugeni C, et al Four delays of child mortality in Rwanda: a mixed methods analysis of verbal social autopsies. BMJ Open. 2019;9(5):e027435 10.1136/bmjopen-2018-027435 31133592 PMC6549629

[19] Valentine N, Verdes-Tennant E, Bonsel G. Health systems' responsiveness and reporting behaviour: Multilevel analysis of the influence of individual-level factors in 64 countries. Social Science & Medicine. 2015;138:152. 26093073 10.1016/j.socscimed.2015.04.022

[20] Salomon JA, Tandon A, Murray CJL. Comparability of Self Rated Health: Cross Sectional Multi-Country Survey Using Anchoring Vignettes. British Medical Journal. 2004;328(7434). 10.1136/bmj.37963.691632.44 14742348 PMC324453

[21] King G, Murray CJL, Salomon JA, Tandon A. Enhancing the Validity and Cross-Cultural Comparability of Measurement in Survey Research. APSR 2004 p. 191–207.

[22] Bostan S, Acuner T, Yilmaz G. Patient (customer) expectations in hospitals. Health policy. 2007;82(1):62–70. 10.1016/j.healthpol.2006.08.005 17028043

[23] Heerwegh D. Mode Differences Between Face-to-Face and Web Surveys: An Experimental Investigation of Data Quality and Social Desirability Effects. International Journal of Public Opinion Research. 2009;21(1):111–21. 10.1093/ijpor/edn054

[24] ComScore. Representativeness of the RIWI Sampling Methodology in the United States. 2014.

[25] Seeman N, Tang S, Brown AD, Ing A. World survey of mental illness stigma. Journal of Affective Disorders. 2016;190:115–21. 10.1016/j.jad.2015.10.011 26496017

[26] Rizo C, Deshpande A, Ing A, Seeman N. A rapid, Web-based method for obtaining patient views on effects and side-effects of antidepressants. Journal of Affective Disorders. 2011;130(1–2):290–3. 10.1016/j.jad.2010.07.027 20705344

[27] Seeman N, Ing A, Rizo C. Assessing and responding in real time to online anti-vaccine sentiment during a flu pandemic. Healthcare quarterly (Toronto, Ont). 2010;13 Spec No:8–15. Epub 2010/10/21. . 20959725 10.12927/hcq.2010.21923

[28] Kruk ME, Paczkowski M, Mbaruku G, de Pinho H, Galea S. Women's preferences for place of delivery in rural Tanzania: a population-based discrete choice experiment. The American Journal of Public Health. 2009;99(9):1666 10.2105/AJPH.2008.146209 19608959 PMC2724466

[29] Kruk ME, Paczkowski MM, Tegegn A, Tessema F, Hadley C, Asefa M, et al Women's preferences for obstetric care in rural Ethiopia: a population-based discrete choice experiment in a region with low rates of facility delivery. J Epidemiol Community Health. 2010;64(11):984–8. 10.1136/jech.2009.087973 19822558

[30] Lungu EA, Guda Obse A, Darker C, Biesma R. What influences where they seek care? Caregivers’ preferences for under-five child healthcare services in urban slums of Malawi: A discrete choice experiment. PLoS ONE. 2018;13(1):e0189940 10.1371/journal.pone.0189940 29351299 PMC5774690

[31] Batbaatar E, Dorjdagva J, Luvsannyam A, Savino MM, Amenta P. Determinants of patient satisfaction: a systematic review. Perspect Public Health. 2017;137(2):89–101. Epub 2016/03/24. 10.1177/1757913916634136 . 27004489

[32] Cheng S-H, Yang M-C, Chiang T-L. Patient satisfaction with and recommendation of a hospital: effects of interpersonal and technical aspects of hospital care. International Journal for Quality in Health Care. 2003;15(4):345–55. 10.1093/intqhc/mzg045 12930050

[33] Fan W, Yan Z. Factors affecting response rates of the web survey: A systematic review. Computers in Human Behavior. 2010;26(2):132–9. 10.1016/j.chb.2009.10.015

[34] Healthcare Access and Quality Index based on mortality from causes amenable to personal health care in 195 countries and territories, 1990–2015: a novel analysis from the Global Burden of Disease Study 2015. Lancet. 2017;390(10091):231–66. Epub 2017/05/23. 10.1016/S0140-6736(17)30818-8 28528753 PMC5528124

[35] Pan B, Woodside AG, Meng F. How Contextual Cues Impact Response and Conversion Rates of Online Surveys. Journal of Travel Research. 2014;53(1):58–68. 10.1177/0047287513484195

[36] Couper MP, Miller PV. Web Survey Methods. Public Opinion Quarterly. 2008;72(5):831–5. 10.1093/poq/nfn066

[37] Manfreda K, Bosnjak M, Berzelak J, Haas I, Vehovar V. Web surveys versus other survey modes: A meta-analysis comparing response rates. International Journal of Market Research. 2008;50(1):79.

[38] Neil S. Results of a New Healthcare Confidence Index. ElectronicHealthcare. 2012;10(4):e5–e11.

[39] Kreuter F, Presser S, Tourangeau R. Social Desirability Bias in CATI, IVR, and Web Surveys. Public Opinion Quarterly. 2008;72(5):847–65. 10.1093/poq/nfn063

[40] Poushter J. Smartphone Ownership and Internet Usage Continues to Climb in Emerging Economies. Pew Research Center [Internet]. 2016 7 16, 2018 Available from: http://www.pewglobal.org/2016/02/22/smartphone-ownership-and-internet-usage-continues-to-climb-in-emerging-economies/. [cited 2019 July 1].

[41] Internet Live Stats. Available from: http://www.internetlivestats.com/. [cited 2018 June 28].

[42] Sénégal Enquête STEPS 2015: Note de synthèse WHO, 2015.

[43] Haagsma JA, Graetz N, Bolliger I, Naghavi M, Higashi H, Mullany EC, et al The global burden of injury: incidence, mortality, disability-adjusted life years and time trends from the Global Burden of Disease study 2013. Injury Prevention. 2016;22(no. 1):3–18. 26635210 10.1136/injuryprev-2015-041616 PMC4752630

[44] Marchant T, Tilley-Gyado RD, Tessema T, Singh K, Gautham M, Umar N, et al Adding content to contacts: measurement of high quality contacts for maternal and newborn health in Ethiopia, north east Nigeria, and Uttar Pradesh, India. PLoS ONE. 2015;10(5):e0126840 Epub 2015/05/23. 10.1371/journal.pone.0126840 26000829 PMC4441429

[45] Das J, Gertler PJ. Variations In Practice Quality In Five Low-Income Countries: A Conceptual Overview. Health Aff. 2007;26(3):w296–309. 10.1377/hlthaff.26.3.w296 17389637

[46] Mohanan M, Hay K, Mor N. Quality Of Health Care In India: Challenges, Priorities, And The Road Ahead. Health Aff (Millwood). 2016;35(10):1753–8. Epub 2016/10/06. 10.1377/hlthaff.2016.0676 . 27702945

[47] Murray CJ, Ortblad KF, Guinovart C, Lim SS, Wolock TM, Roberts DA, et al Global, regional, and national incidence and mortality for HIV, tuberculosis, and malaria during 1990–2013: a systematic analysis for the Global Burden of Disease Study 2013. Lancet. 2014;384(9947):1005–70. Epub 2014/07/26. 10.1016/S0140-6736(14)60844-8 25059949 PMC4202387

[48] Ministry of Health Kingdom of Morocco U. Reducing Maternal Mortality in Morocco: Sharing Experience and Sustaining Progress. 2011.

[49] Republic of Kenya Ministry of Devolution and Planning. Millenium Development Goals Status Report 2013. 2014.

[50] Irimu G, Ogero M, Mbevi G, Kariuki C, Gathara D, Akech S, et al Tackling health professionals’ strikes: an essential part of health system strengthening in Kenya. BMJ Global Health. 2018;3(6):e001136 10.1136/bmjgh-2018-001136 30588346 PMC6278918

[51] Rankinen S, Salanterä S, Heikkinen K, Johansson K, Kaljonen A, Virtanen H, et al Expectations and received knowledge by surgical patients. International journal for quality in health care: journal of the International Society for Quality in Health Care. 2007;19(2):113 10.1093/intqhc/mzl075 17277008

[52] Srivastava A, Avan BI, Rajbangshi P, Bhattacharyya S. Determinants of women’s satisfaction with maternal health care: a review of literature from developing countries. BMC Pregnancy and Childbirth. 2015;15:1–12. 10.1186/s12884-015-0429-z 25928085 PMC4417271

[53] Oladapo OT, Osiberu MO. Do sociodemographic characteristics of pregnant women determine their perception of antenatal care quality? Matern Child Health J. 2009;13(4):505–11. Epub 2008/07/17. 10.1007/s10995-008-0389-2 . 18629621

[54] Larson E, Hermosilla S, Kimweri A, Mbaruku GM, Kruk ME. Determinants of perceived quality of obstetric care in rural Tanzania: a cross-sectional study. BMC Health Services Research. 2014;14(1). 10.1186/1472-6963-14-483 25326007 PMC4283093

[55] Sharma J, Leslie HH, Kundu F, Kruk ME. Poor quality for poor women? Inequities in the quality of antenatal and delivery care in Kenya. PLoS ONE. 2017;12(1):e0171236 10.1371/journal.pone.0171236 28141840 PMC5283741

[56] Sen A. Health: perception versus observation. BMJ. 2002;324(7342):860 10.1136/bmj.324.7342.860 11950717 PMC1122815

[57] Bertakis KD, Azari R, Helms LJ, Callahan EJ, Robbins JA. Gender differences in the utilization of health care services. The Journal of family practice. 2000;49(2):147–52. Epub 2000/03/16. . 10718692

[58] Langer A, Meleis A, Knaul FM, Atun R, Aran M, Arreola-Ornelas H, et al Women and Health: the key for sustainable development. The Lancet. 2015;386(9999):1165–210. 10.1016/s0140-6736(15)60497-4 26051370

[59] Souza JP, Gülmezoglu AM, Vogel J, Carroli G, Lumbiganon P, Qureshi Z, et al Moving beyond essential interventions for reduction of maternal mortality (the WHO Multicountry Survey on Maternal and Newborn Health): a cross-sectional study. Lancet. 2013;381(9879):1747–55. 10.1016/S0140-6736(13)60686-8 23683641

[60] Bohren MA, Vogel JP, Hunter EC, Lutsiv O, Makh SK, Souza JP, et al The Mistreatment of Women during Childbirth in Health Facilities Globally: A Mixed-Methods Systematic Review. PLoS Med. 2015;12 10.1371/journal.pmed.1001847 26126110 PMC4488322

[61] Leslie HH, Spiegelman D, Zhou X, Kruk ME. Service readiness of health facilities in Bangladesh, Haiti, Kenya, Malawi, Namibia, Nepal, Rwanda, Senegal, Uganda and the United Republic of Tanzania. Bull World Health Organ. 2017;95(11). 10.2471/BLT.17.191916 PMC5677617 29147054

[62] Victoor A, Delnoij Diana M, Friele Roland D, Rademakers Jany J. Determinants of patient choice of healthcare providers: a scoping review. BMC Health Services Research. 2012;12(1):272 10.1186/1472-6963-12-272 22913549 PMC3502383

[63] Björkman M, Svensson J. Power to the People: Evidence from a Randomized Field Experiment on Community-Based Monitoring in Uganda. Q J Econ. 2009;124(2):735–69. 10.1162/qjec.2009.124.2.735

[64] Prost A, Colbourn T, Seward N, Azad K, Coomarasamy A, Copas A, et al Women's groups practising participatory learning and action to improve maternal and newborn health in low-resource settings: a systematic review and meta-analysis. The Lancet. 2013;381(9879, 18–24):1736–46. 10.1016/S0140-6736(13)60685-6 PMC3797417 23683640

[65] Timian A, Rupcic S, Kachnowski S, Luisi P. Do Patients “Like” Good Care? Measuring Hospital Quality via Facebook. American Journal of Medical Quality. 2013;28(5):374–82. 10.1177/1062860612474839 23378059

[66] Islam MM, Touray M, Yang H-C, Poly TN, Nguyen P-A, Li Y-CJ, et al E-Health Literacy and Health Information Seeking Behavior Among University Students in Bangladesh. Studies in health technology and informatics. 2017;245:122 29295065

[67] Oliveira-Ciabati L, Vieira CS, Franzon ACA, Alves D, Zaratini FS, Braga GC, et al PRENACEL—a mHealth messaging system to complement antenatal care: a cluster randomized trial. Reprod Health. 2017;14(1):146 Epub 2017/11/09. 10.1186/s12978-017-0407-1 29116028 PMC5678588

