# Supplementary material for: Expectations of healthcare quality: A cross-sectional study of internet users in 12 low- and middle-income countries
Source: PLoS Med. 2019 Aug 7;16(8):e1002879. doi: 10.1371/journal.pmed.1002879 (PMC6685603; doi:10.1371/journal.pmed.1002879)
Supplement: S1 Appendix — (DOCX) [file pmed.1002879.s001.docx]

**Expectations of healthcare quality: a cross-sectional study of internet users in 12 low- and middle-income countries**

*S1 Appendix: Survey instrument*

**Note:** Programming instructions are in red, response options are in italics. All questions are asked to all respondents unless otherwise noted.

[X] University is studying how to make health care better in [**Country name**]. Please help us by participating in the short voluntary, anonymous survey. Your identity is completely protected.

**Question 1.**

What is your age and gender?

**Age/gender selection**

**Question 2.**

In general, would you say your health is…

*Excellent*

*Very good*

*Good*

*Fair*

*Poor*

**Question 3.**

In general, how would you rate your mental health, including your mood?

*Excellent*

*Very good*

*Good*

*Fair*

*Poor*

**Question 4.**

Where do you live?

*City*

*Town*

*Village*

**Question 5.**

What is the highest level of education you have completed?

*No formal schooling*

*Some primary school*

*Primary school completed*

*Some secondary or high school*

*Secondary or high school completed*

*Some college or university*

*Completed college or university*

**Change names in Question 7 and 8 according to country:**

| Country | Name |
| --- | --- |
| South Africa | Junior |
| Nigeria | Femi |
| Kenya | John |
| Senegal | Babacar |
| Ghana | Afram |
| Mexico | Juan |
| Argentina | Juan |
| India | Krishna |
| China | Zhang Wei |
| Indonesia | Panji |
| Lebanon | Adam |
| Morocco | Youssef |

**Question 7.**

[**Anthony**] is a 45-year old man with high blood pressure who needs a regular check-up. At the health facility, the nurse

DOES: -Greet him and introduce herself -Change his medications

DOES NOT: -Ask about his symptoms -check his blood pressure

How would you rate the overall quality of care that [Anthony] received?

*Poor*

*Fair*

*Good*

*Very Good*

*Excellent*

**Question 8.**

**Randomize receipt of one of the five vignettes below:**

1. [**Anthony**] is a 45-year old man with high blood pressure who needs a regular check-up. At the health facility, the nurse…

DOES: -check his blood pressure -Change his medications

DOES NOT: -Greet him and introduce herself -Ask about his symptoms

1. [**Anthony**] is a 45-year old man with high blood pressure who needs a regular check-up. At the health facility, the nurse…

DOES: -Change his medications

DOES NOT: -Greet him and introduce herself -Ask about his symptoms -check his blood pressure

1. [**Anthony**] is a 45-year old man who has hurt his arm in a motorcycle accident. 
   At the health facility, the nurse...

DOES: -Greet him and introduce herself -Wrap his arm with a bandage

DOES NOT: -Ask about his arm -Examine his arm

1. [**Anthony**] is a 45-year old man who has hurt his arm in a motorcycle accident. 
   At the health facility, the nurse...

DOES: -Examine his arm - Wrap his arm with a bandage

DOES NOT: -Greet him and introduce herself -Ask about his arm

1. [**Anthony**] is a 45-year old man who has hurt his arm in a motorcycle accident. 
   At the health facility, the nurse...

DOES: -Wrap his arm with a bandage

DOES NOT: -Greet him and introduce herself -Ask about his arm -Examine his arm

How would you rate the overall quality of care that [**Anthony**] received?

*Poor*

*Fair*

*Good*

*Very Good*

*Excellent*

**Question 9.**

In the **past year**, how many times did you go to a clinic, health center, or hospital to receive health care for yourself? (Please do not include any times you stayed overnight.)

*0*

*1*

*2*

*3*

*4*

*5*

*6*

*7*

*8*

*9*

*10+*

**NOTE: If Q9 = 0, skip to Q19**

**Question 10.**

What was the main reason for your last visit to a health care facility? (Excluding overnight hospital stays)

*Emergency, accident, or injury*

*Pregnancy or delivery related*

*Check-up, annual exam, or preventive care*

*Chronic existing problem*

*New health problem or question*

**Question 11.**

Who managed the last facility that you visited for care (excluding overnight hospital stays)?

*Government*

*Private*

**Show prompt below for Questions 12-16**

| Thinking about your last visit to a health care facility (not including overnight stays), how would you rate the following: |
| --- |

**Randomize order of Questions 12-16**

**Question 12.**

The length of time you waited before you were seen.

*Poor*

*Fair*

*Good*

*Very Good*

*Excellent*

**Question 13.**

Whether the provider listened carefully to you.

*Poor*

*Fair*

*Good*

*Very Good*

*Excellent*

**Question 14.**

Provider’s medical knowledge and skills.

*Poor*

*Fair*

*Good*

*Very good*

*Excellent*

**Question 15.**

The level of respect the provider showed you.

*Poor*

*Fair*

*Good*

*Very good*

*Excellent*

**Question 16.**

The amount of time the provider spent with you in the visit.

*Poor*

*Fair*

*Good*

*Very good*

*Excellent*

**Question 17.**

Overall, taking everything into account, how would you rate the quality of care you received?

*Poor*

*Fair*

*Good*

*Very good*

*Excellent*

**Question 18.**

Overall, thinking about your entire visit, how satisfied were you with the care you received?

*Very dissatisfied*

*Somewhat dissatisfied*

*Somewhat satisfied*

*Very satisfied*

**Question 19.**

In the last year, was there a time when you had a medical problem and needed medical attention, but you did not visit a health facility?

*Yes*

*No*

*No medical problems*

**NOTE: If Q19=No or No medical problems, skip to Q21**

**Question 20.**

What is the main reason you did not visit a health facility?

What there another reason you did not visit a health facility?

What there another reason you did not visit a health facility?

**Randomize list of response options below. Allow respondents to choose option at a time, re-randomize, and ask second question.**

*None of the above*

*Too far/no transport*

*Costs are too high*

*Drugs/supplies not available*

*Health care providers lack knowledge/skills*

*Wait times are too long*

*Health care staff are disrespectful*

**Question 21.**

Have you ever been discriminated against, hassled, or made to feel inferior by a health provider/staff for any of these reasons?

- Poverty
- Religion
- Ethnicity, Language
- Immigration, Migrant status
- Because of your gender
- Type of Illness
- Education
- Romantic or Sexual attraction to someone of the same sex
- Any other reason

*Yes*

*No*

**Question 22.**

You mentioned that you were discriminated against by health providers/staff. What were the reasons? Choose as many as apply.

*Poverty*

*Religion*

*Ethnicity, Language*

*Immigration, Migrant Status*

*Education*

*Romantic or sexual attraction to someone of the same sex*

*Type of illness*

*Because I am a man/woman*

*Other*

**Question 23.**

How confident are you that if you become very sick tomorrow, you would be able to receive effective treatment from the health system?

*Not at all confident*

*Not very confident*

*Somewhat confident*

*Very confident*

**Question 24.**

With which statement do you agree most?

*Our health care system has so much wrong with it that we need* ***to completely rebuild it****.*

*There are some good things in our health care system, but* ***major changes*** *are needed to make it work better.*

*On the whole, the system works pretty well and only* ***minor changes*** *are necessary to make it work better.*

**Question 25.**

Do you have health insurance? (Any service, either from the government or a private company, that pays for some or all of your health care costs)

*Yes*

*No*

**End survey with note below:**

| Thank you for participating in this survey. We appreciate your time. |
| --- |
