# Supplementary material for: Expectations of healthcare quality: A cross-sectional study of internet users in 12 low- and middle-income countries
Source: PLoS Med. 2019 Aug 7;16(8):e1002879. doi: 10.1371/journal.pmed.1002879 (PMC6685603; doi:10.1371/journal.pmed.1002879)

**Expectations of healthcare quality: a cross-sectional study of internet users in 12 low- and middle-income countries**

*S2 Appendix: Survey instrument screen shot*


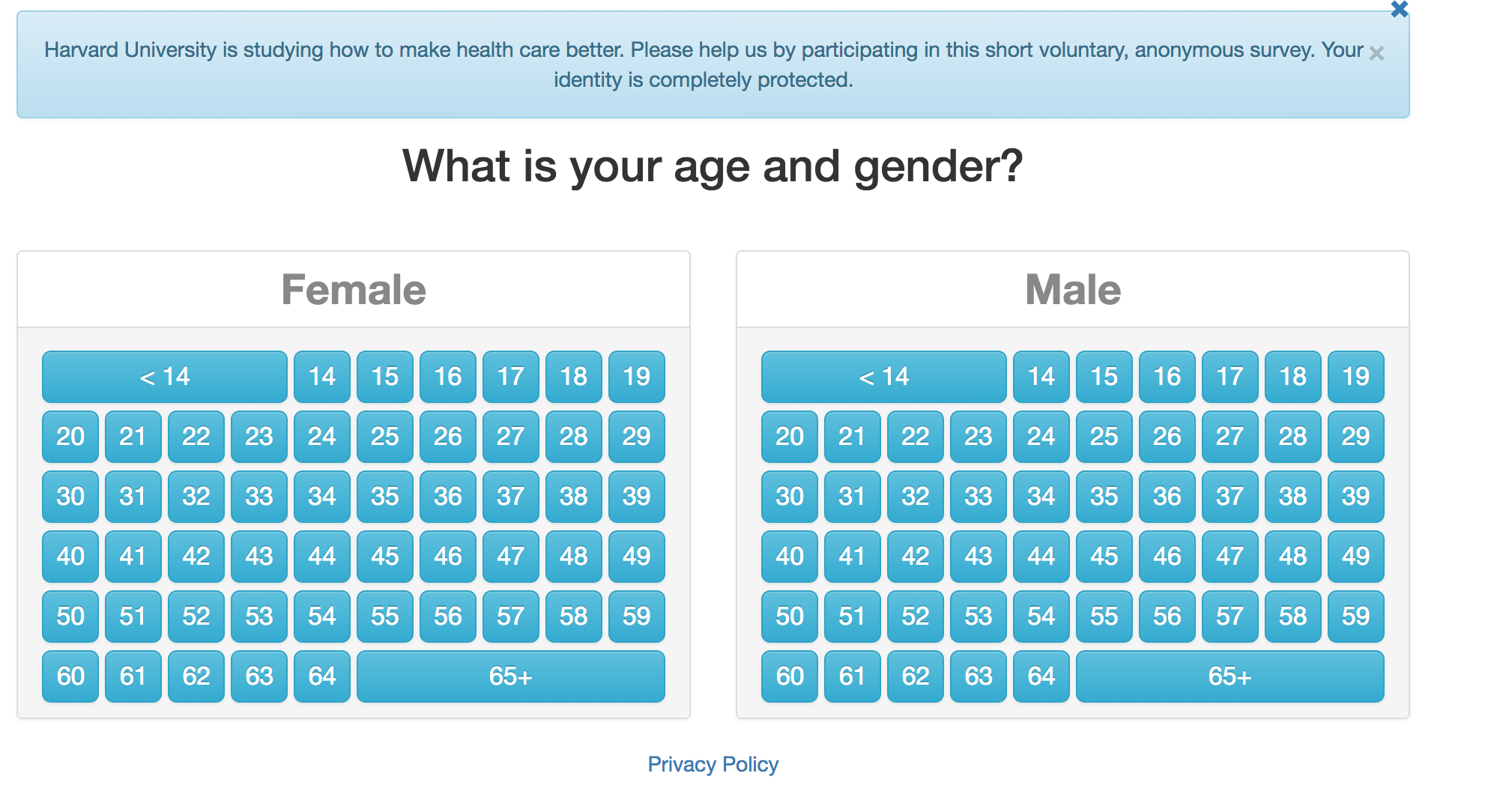

Supplement: S2 Appendix — (DOCX) [file pmed.1002879.s002.docx]
