# Supplementary material for: Expectations of healthcare quality: A cross-sectional study of internet users in 12 low- and middle-income countries
Source: PLoS Med. 2019 Aug 7;16(8):e1002879. doi: 10.1371/journal.pmed.1002879 (PMC6685603; doi:10.1371/journal.pmed.1002879)
Supplement: S3 Appendix — (DOCX) [file pmed.1002879.s003.docx]

**Expectations of healthcare quality: a cross-sectional study of internet users in 12 low- and middle-income countries**

*S3 Appendix: Internet penetration rates in survey countries*

| **Country** | **Internet penetration (2016)** |
| --- | --- |
| Senegal | 23.4% |
| Ghana | 28.4% |
| Kenya | 45.0% |
| India | 34.8% |
| Nigeria | 46.1% |
| Morocco | 57.6% |
| Indonesia | 20.4% |
| South Africa | 52.0% |
| Lebanon | 75.9% |
| China | 52.2% |
| Mexico | 45.1% |
| Argentina | 69.2% |

Caption: Data source is [www.internetlivestats.com](http://www.internetlivestats.com/)
