# Supplementary material for: Expectations of healthcare quality: A cross-sectional study of internet users in 12 low- and middle-income countries
Source: PLoS Med. 2019 Aug 7;16(8):e1002879. doi: 10.1371/journal.pmed.1002879 (PMC6685603; doi:10.1371/journal.pmed.1002879)
Supplement: S4 Appendix — (DOCX) [file pmed.1002879.s004.docx]

**Expectations of healthcare quality: a cross-sectional study of internet users in 12 low- and middle-income countries**

*S4 Appendix: Experiences and perceptions of healthcare quality in LMIC*

*Internet survey Analysis Plan*

**Research Question**

What do people think of their healthcare in 12 LMICs?

1. What are expectations of healthcare in LMICs across socio-demographic and contextual factors?
2. What are experiences of healthcare in LMICs across socio-demographic and contextual factors?
   1. How do people rate their care (e.g. quality and satisfaction)?
   2. Do these ratings change when adjusting for expectations of care?
3. How do people rate their health systems?
   1. Do these ratings change when adjusting for met expectations of care?

**Hypotheses**

Logit (Vignettes) = α + β_1_(demograph) + β_2_(utilization) + β_3_ (health status) + i.country +  e

Research questions: 1

Outcome: valuations of care (expectations)

*Expectations of care are shaped by demographic factors, country and health system utilization history and health status. Lower expectations are correlated with lower socioeconomic status, country wealth. In wealthy countries with higher quality health systems, utilization is correlated with higher expectations. In poor countries with lower quality health systems, utilization is correlated with lower expectations. Utilization is an effect modifier. Can we fully predict expectations with demographics? Is what we can’t predict explained by the country dummy and the quality of the country’s health system?*

Logit (Quality rating_q1-6_) = α + β_1_(vignettes) + β_2_(reason for visit) + β_3_ (demograph) + i.country +  e

Research questions: 2a, 2b

Outcome: experience of care

*Rating of quality of experience is shaped by your expectations of care.*

*Low expectations of care and high quality ratings are correlated (but can’t measure actual quality, possible that people with high expectations are accessing objectively better care).*

Logit (Discrimination) = α + β_1_(demograph) + β_2_ (health status) + i.country +  e

Research questions: 2

Outcome: discrimination

Logit (System quality) = α + β_1_(quality rating modified by vignettes) + β_2_(demograph) + β_3_ (utilization)+ β_4_(health status) + i.country + e

Logit (Confidence) = α + β_1_(quality rating modified by vignettes) + β_2_(demograph) + β_3_ (utilization) + β_4_(health status) + i.country +e

Logit (Satisfaction) = α + β_1_(quality rating modified by vignettes) + β_2_(demograph) + β_3_ (utilization) + β_4_(health status) + i.country + e

Research questions: 2a, 3, 3a

Outcome: assessment of care

*System quality rating, confidence in system and satisfaction with care are all correlated with your met expectations, not just your quality rating. Expectations are an effect modifier*
