# Supplementary material for: Expectations of healthcare quality: A cross-sectional study of internet users in 12 low- and middle-income countries
Source: PLoS Med. 2019 Aug 7;16(8):e1002879. doi: 10.1371/journal.pmed.1002879 (PMC6685603; doi:10.1371/journal.pmed.1002879)
Supplement: S5 Appendix — (DOCX) [file pmed.1002879.s005.docx]

**Expectations of healthcare quality: a cross-sectional study of internet users in 12 low- and middle-income countries**

*S5 Appendix: Survey Languages*

| **Country** | **Survey Language** |
| --- | --- |
| Senegal | Senegalese French |
| Ghana | English |
| Kenya | English |
| India | English^b^ |
| Nigeria | English |
| Morocco | Moroccan Arabic |
| Indonesia | Bahasa |
| S. Africa | English |
| Lebanon | Lebanese Arabic |
| China | Chinese |
| Mexico | Mexican Spanish |
| Argentina | Argentine Spanish |

Caption: Languages were chosen based on most commonly spoken language in country. Previous experience with internet surveys in India by the RIWI Corp showed that, when given a choice, Indian internet respondents overwhelmingly selected English
