# Supplementary material for: Expectations of healthcare quality: A cross-sectional study of internet users in 12 low- and middle-income countries
Source: PLoS Med. 2019 Aug 7;16(8):e1002879. doi: 10.1371/journal.pmed.1002879 (PMC6685603; doi:10.1371/journal.pmed.1002879)
Supplement: S6 Appendix — (DOCX) [file pmed.1002879.s006.docx]

**Expectations of healthcare quality: a cross-sectional study of internet users in 12 low- and middle-income countries**

*S6 Appendix: Expanded rationale for methods*

The RDIT^tm^ methodology has been used in healthcare to explore mental illness stigma, anti-vaccination beliefs and healthcare utilization behavior [1,2]. It produces reliable results over time. In a study in India, RDIT^tm^ was used to distribute a single short survey every month for 21 months. Results were consistent over the study period and standard errors were low [1-3]. RDIT^tm^ generates a study sample that closely approximates internet users at large because the interception method – entering non-existent site names – is common. This was illustrated in an analysis comparing the geographic distribution, internet service providers, and internet use characteristics (e.g. number of sites visited) of RDIT^tm^-generated samples to internet users nationally in the United States. The two samples were nearly identical on every measure with correlations between .915 and .997. Though quite different from our study countries, the United States is a particularly good country in which to do this analysis because of the large amount of information available on internet users [4].

We selected the RDIT^tm^ methodology because it has important advantages over other internet surveying methods such as internet panels and email-based sampling [5,6]. Participants in internet panels become habituated to taking surveys, leading to inattentiveness and jadedness [7,8]. In contrast, RDIT^tm^-generated samples tend to be composed of casual respondents. In a study of over 50,000 internet users sampled through this method, almost half (47%) had never taken a survey before. Email can also be used to invite participants to an internet survey, but these solicitations may be filtered out by email servers, respondents cannot participate anonymously, and samples tend to over-represent habitual respondents [5].

**References**

1. Seeman N, Ing A, Rizo C. Assessing and responding in real time to online anti-vaccine sentiment during a flu pandemic. Healthcare quarterly (Toronto, Ont). 2010;13 Spec No:8-15. Epub 2010/10/21. PubMed PMID: 20959725.

2. Seeman N, Tang S, Brown AD, Ing A. World survey of mental illness stigma. Journal of Affective Disorders. 2016;190:115-21. doi: 10.1016/j.jad.2015.10.011.

3. Rizo C, Deshpande A, Ing A, Seeman N. A rapid, Web-based method for obtaining patient views on effects and side-effects of antidepressants. Journal of Affective Disorders. 2011;130(1-2):290-3. doi: 10.1016/j.jad.2010.07.027.

4. ComScore. Representativeness of the RIWI Sampling Methodology in the United States. 2014.

5. Schonlau M, Couper MP. Options for Conducting Web Surveys. Statist Sci. 2017;32(2):279-92. doi: 10.1214/16-STS597.

6. Erens B, Burkill S, Copas A, Couper M, Conrad F. How well do volunteer web panel surveys measure sensitive behaviours in the general population, and can they be improved? A comparison with the third British National Survey of Sexual Attitudes & Lifestyles (Natsal-3). Lancet. 2013;382:34-.

7. TOEPOEL VD, Marcel; VAN SOEST, Arthur. Relating Question Type to Panel Conditioning: Comparing Trained and Fresh Respondents. Survey Research Methods. 2009;3(2).

8. Murphy L., Pospichal L., Liebenson D., Meerkamper E. GRIT Consumer Participation in Research Report: A Global Study of Habitual vs. Non-Habitual Survey Takers. New York, NY: Greenbook and RIWI, 2014.
