# Supplementary material for: Expectations of healthcare quality: A cross-sectional study of internet users in 12 low- and middle-income countries
Source: PLoS Med. 2019 Aug 7;16(8):e1002879. doi: 10.1371/journal.pmed.1002879 (PMC6685603; doi:10.1371/journal.pmed.1002879)
Supplement: S7 Appendix — (DOCX) [file pmed.1002879.s007.docx]

**Expectations of healthcare quality: a cross-sectional study of internet users in 12 low- and middle-income countries**

*S7 Appendix: Conceptual framework for expectations of quality*


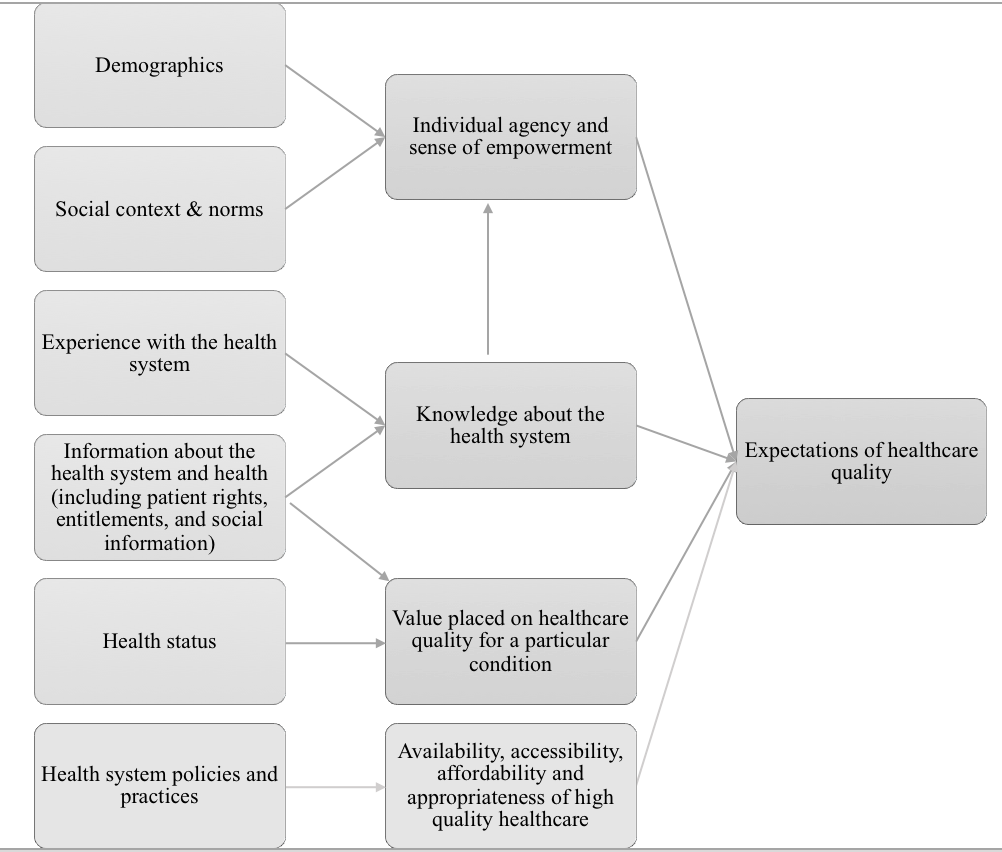


*Caption: The framework is informed by the Andersen model of healthcare-seeking behavior, but is adapted to focus on preferential use of high quality care, rather than access to care more generally. It also draws on models from high-income settings and ideas from the social sciences on health perceptions and experiences.[10-14] Five domains are theorized to contribute to three mechanisms that shape expectations of healthcare. Demographics interact with the social context and norms around healthcare to form the first mechanism, individual agency and sense of empowerment. Broadly, socio-economic vulnerability is thought to lead to lower agency and sense of empowerment. Personal experience and information about the health system shape the second mechanism, knowledge about the health system. This knowledge also increases a sense of empowerment. Information about the health system in addition to the specific health condition or health status forms the third mechanism, value placed on quality of healthcare. Conditions that are perceived to be more serious or dangerous are thought to lead to a higher value placed on high quality care. The three mechanisms, in combination with the supply of healthcare, i.e. the availability, accessibility, affordability and appropriateness of quality care, are proposed to influence expectations of healthcare quality.*
