# Supplementary material for: Expectations of healthcare quality: A cross-sectional study of internet users in 12 low- and middle-income countries
Source: PLoS Med. 2019 Aug 7;16(8):e1002879. doi: 10.1371/journal.pmed.1002879 (PMC6685603; doi:10.1371/journal.pmed.1002879)
Supplement: S8 Appendix — (DOCX) [file pmed.1002879.s008.docx]

**Expectations of healthcare quality: a cross-sectional study of internet users in 12 low- and middle-income countries**

*S8 Appendix: Country demographic data*

| **Socio-demographics, mid-year 2017** | | |  | |  | |  |  | |  | |  | |  | |  |  | |  | |  |  |  |
| --- | --- | --- | --- | --- | --- | --- | --- | --- | --- | --- | --- | --- | --- | --- | --- | --- | --- | --- | --- | --- | --- | --- | --- |
|  | Argentina | China | | Ghana | | India | | | Indonesia | | Kenya | | Lebanon | | Mexico | | | Morocco | | Nigeria | | Senegal | South Africa |
| Total population | 44293293 | 1379302771 | | 27499924 | | 1281935911 | | | 260580739 | | 47615739 | | 6229794 | | 124574795 | | | 33986655 | | 198346055 | | 14668522 | 54841552 |
|  |  |  | |  | |  | | |  | |  | |  | |  | | |  | |  | |  |  |
| Male | 49% | 51% | | 49% | | 52% | | | 50% | | 50% | | 50% | | 49% | | | 49% | | 50% | | 48% | 49% |
|  |  |  | |  | |  | | |  | |  | |  | |  | | |  | |  | |  |  |
| Urban population | 92% | 58% | | 55% | | 34% | | | 55% | | 27% | | 88% | | 80% | | | 61% | | 49% | | 44% | 66% |
|  |  |  | |  | |  | | |  | |  | |  | |  | | |  | |  | |  |  |
| Primary Education or less | 44% | 35% | | 46% | | 62% | | | 51% | | 71% | | 46% | | 41% | | | 70% | | 54% | | 86% | 23% |
|  |  |  | |  | |  | | |  | |  | |  | |  | | |  | |  | |  |  |
| Age: 18-29 | 18% | 18% | | 20% | | 21% | | | 19% | | 21% | | 20% | | 21% | | | 20% | | 20% | | 22% | 23% |
|  |  |  | |  | |  | | |  | |  | |  | |  | | |  | |  | |  |  |
| Age: 30-49 | 27% | 31% | | 23% | | 28% | | | 29% | | 23% | | 31% | | 27% | | | 29% | | 20% | | 20% | 28% |
|  |  |  | |  | |  | | |  | |  | |  | |  | | |  | |  | |  |  |
| Age: 50+ | 26% | 28% | | 13% | | 19% | | | 21% | | 10% | | 20% | | 20% | | | 20% | | 10% | | 10% | 17% |
| Caption: Total population, gender, and age demographic information from 2017 was accessed through the United States Census Bureau’s International Data Base.  The United States Central Intelligence Agency database was used to create residence data. Educational attainment data for those over 25 years old was accessed through the UNESCO Institute for Statistics UIS.Stat tool, with some exceptions. Data related to Nigeria is from the Demographic and Health Survey and data related to Morocco was accessed through the UNESCO High Commission for Education | | | | | | | | | | | | | | | | | | | | | | | |
