# Supplementary material for: Expectations of healthcare quality: A cross-sectional study of internet users in 12 low- and middle-income countries
Source: PLoS Med. 2019 Aug 7;16(8):e1002879. doi: 10.1371/journal.pmed.1002879 (PMC6685603; doi:10.1371/journal.pmed.1002879)
Supplement: S9 Appendix — (DOCX) [file pmed.1002879.s009.docx]

**Expectations of healthcare quality: a cross-sectional study of internet users in 12 low- and middle-income countries**

*S9 Appendix: Weights*

Weights were created for age, gender, urban/rural residence and educational attainment using a ranking algorithm designed to approximate population characteristics in each of the 12 study countries. United States [Census Bureau](https://www.census.gov/data-tools/demo/idb/informationGateway.php) estimates from 2017 were used to create age and gender targets [1]. The United States Central Intelligence Agency database was used to create residence targets. The education targets for most countries were created based on educational attainment data from UNESCO Institute for Statistics data. Nigeria’s targets were made based on Demographic and Health Survey data and Moroccan targets were created using data from the High Commission for Education targets for Nigeria were created based on data from the Demographic and Health Survey conducted by the National Population Commission for the Plan of Morocco.

**References**

1. United States Census Bureau. International database 2017.
