# Supplementary material for: Expectations of healthcare quality: A cross-sectional study of internet users in 12 low- and middle-income countries
Source: PLoS Med. 2019 Aug 7;16(8):e1002879. doi: 10.1371/journal.pmed.1002879 (PMC6685603; doi:10.1371/journal.pmed.1002879)
Supplement: S10 Appendix — (DOCX) [file pmed.1002879.s010.docx]

**Expectations of healthcare quality: a cross-sectional study of internet users in 12 low- and middle-income countries**

*S10 Appendix: Ordered logistic regression*

|  | **Blood pressure visit; poor technical quality** | | | **Accident visit; poor technical quality** | | | **Blood pressure visit; poor interpersonal quality** | | | **Accident visit; poor interpersonal quality** | | |
| --- | --- | --- | --- | --- | --- | --- | --- | --- | --- | --- | --- | --- |
|  | **(n=17996)** | | | **(n=3640)** | | | **(n=3541)** | | | **(n=3667)** | | |
|  | Adjusted Odds Ratio | 95% CI | *P*-value | Adjusted Odds Ratio | 95% CI | *P*-value | Adjusted Odds Ratio | 95% CI | *P*-value | Adjusted Odds Ratio | 95% CI | *P*-value |
| Male gender | 1.27 | [1.18,1.38] | <.001 | 1.22 | [1.04,1.44] | 0.02 | 1.30 | [1.08,1.56] | 0.01 | 1.19 | [1.03,1.38] | 0.02 |
| Age | 1.00 | [1.00,1.01] | 0.06 | 1.01 | [1.00,1.01] | 0.00 | 1.00 | [0.99,1.01] | 0.59 | 1.00 | [1.00,1.01] | 0.57 |
| Educational attainment (ref: completed college or university) | | | | | |  |  |  |  |  |  |  |
| -Some college or university | 1.13 | [0.99,1.29] | 0.07 | 1.06 | [0.89,1.26] | 0.52 | 1.13 | [0.94,1.36] | 0.20 | 1.10 | [0.85,1.42] | 0.48 |
| -Secondary or high school completed | 1.30 | [1.16,1.44] | <.001 | 1.32 | [1.08,1.60] | 0.01 | 1.29 | [1.02,1.62] | 0.03 | 1.18 | [0.96,1.46] | 0.12 |
| -Some secondary or high school | 1.64 | [1.43,1.87] | <.001 | 1.65 | [1.28,2.13] | <.001 | 1.49 | [1.21,1.83] | <.001 | 1.71 | [1.32,2.21] | <.001 |
| -Primary school completed | 1.97 | [1.60,2.42] | <.001 | 1.74 | [1.43,2.12] | <.001 | 2.60 | [1.89,3.59] | <.001 | 1.39 | [1.07,1.80] | 0.01 |
| -Some primary school | 2.32 | [1.72,3.13] | <.001 | 3.55 | [2.08,6.08] | <.001 | 1.88 | [1.10,3.21] | 0.02 | 1.56 | [0.94,2.59] | 0.08 |
| -No formal schooling | 2.11 | [1.74,2.57] | <.001 | 1.73 | [1.23,2.44] | 0.00 | 2.15 | [1.62,2.85] | <.001 | 1.47 | [0.97,2.22] | 0.07 |
| Rural residence | 1.00 | [0.90,1.12] | 0.95 | 1.05 | [0.88,1.26] | 0.57 | 0.95 | [0.79,1.15] | 0.62 | 0.93 | [0.80,1.09] | 0.36 |
| Self-reported health (ref: poor) |  |  |  |  |  |  |  |  |  |  |  |  |
| -Fair | 1.36 | [1.02,1.81] | 0.04 | 1.62 | [1.07,2.43] | 0.02 | 1.77 | [1.06,2.94] | 0.03 | 1.26 | [0.86,1.84] | 0.24 |
| -Good | 2.06 | [1.49,2.85] | <.001 | 2.70 | [1.58,4.60] | <.001 | 2.94 | [1.80,4.80] | <.001 | 1.76 | [1.21,2.55] | 0.00 |
| -Very good | 2.87 | [1.89,4.35] | <.001 | 3.44 | [1.79,6.62] | <.001 | 4.73 | [2.70,8.29] | <.001 | 2.94 | [1.93,4.48] | <.001 |
| -Excellent | 6.89 | [4.21,11.30] | <.001 | 9.85 | [4.88,19.88] | <.001 | 10.86 | [5.40,21.87] | <.001 | 8.79 | [5.45,14.17] | <.001 |
| Number outpatient visits in past year (ref: none) | 1.03 | [1.01,1.05] | 0.01 | 1.05 | [1.03,1.08] | <.001 | 1.02 | [0.99,1.06] | 0.24 | 1.04 | [1.01,1.07] | 0.01 |
| Ever experienced discrimination (ref: no discrimination) | 1.43 | [1.26,1.62] | <.001 | 1.29 | [1.13,1.47] | <.001 | 1.36 | [1.13,1.64] | 0.00 | 1.24 | [1.04,1.47] | 0.01 |
| Country (ref: Senegal) |  |  |  |  |  |  |  |  |  |  |  |  |
| Ghana | 1.16 | [1.13,1.19] | <.001 | 1.73 | [1.62,1.84] | <.001 | 0.79 | [0.73,0.85] | <.001 | 1.14 | [1.07,1.22] | <.001 |
| Kenya | 0.88 | [0.84,0.91] | <.001 | 1.15 | [1.11,1.19] | <.001 | 0.58 | [0.52,0.65] | <.001 | 0.78 | [0.73,0.82] | <.001 |
| India | 2.04 | [1.90,2.19] | <.001 | 2.12 | [1.95,2.30] | <.001 | 1.95 | [1.72,2.20] | <.001 | 2.63 | [2.35,2.95] | <.001 |
| Nigeria | 1.35 | [1.30,1.39] | <.001 | 1.60 | [1.50,1.69] | <.001 | 1.10 | [1.02,1.18] | 0.01 | 1.32 | [1.24,1.41] | <.001 |
| Morocco | 0.74 | [0.73,0.76] | <.001 | 0.90 | [0.86,0.94] | <.001 | 0.43 | [0.40,0.46] | <.001 | 0.65 | [0.61,0.69] | <.001 |
| Indonesia | 1.65 | [1.51,1.79] | <.001 | 1.76 | [1.58,1.96] | <.001 | 1.37 | [1.23,1.52] | <.001 | 1.58 | [1.43,1.76] | <.001 |
| South Africa | 1.27 | [1.22,1.32] | <.001 | 1.28 | [1.19,1.38] | <.001 | 1.12 | [1.07,1.18] | <.001 | 0.98 | [0.90,1.07] | 0.63 |
| Lebanon | 1.61 | [1.51,1.70] | <.001 | 1.79 | [1.64,1.95] | <.001 | 1.57 | [1.49,1.66] | <.001 | 1.65 | [1.51,1.81] | <.001 |
| China | 1.71 | [1.62,1.81] | <.001 | 1.59 | [1.48,1.72] | <.001 | 1.44 | [1.34,1.56] | <.001 | 1.41 | [1.32,1.50] | <.001 |
| Mexico | 1.22 | [1.16,1.27] | <.001 | 1.38 | [1.30,1.47] | <.001 | 0.92 | [0.84,1.01] | 0.07 | 1.19 | [1.11,1.27] | <.001 |
| Argentina | 1.17 | [1.10,1.25] | <.001 | 1.29 | [1.17,1.43] | <.001 | 1.13 | [1.02,1.26] | 0.03 | 1.39 | [1.26,1.54] | <.001 |

*Caption: These results are from multivariable ordered logistic regressions. Standard errors are robust and clustered by country. Data is unweighted. The outcome variable includes 5 response categories: Poor, fair, good, very good, excellent. The reference response category is “poor.” The prompt for number of visits was: “In the past year, how many times did you go to a clinic, health center, or hospital to receive health care for yourself? (Please do not include any times you stayed overnight.)”. The question regarding discrimination was: “Have you ever been discriminated against, hassled, or made to feel inferior by a health provider/staff for any of these reasons?”*
