# Supplementary material for: Expectations of healthcare quality: A cross-sectional study of internet users in 12 low- and middle-income countries
Source: PLoS Med. 2019 Aug 7;16(8):e1002879. doi: 10.1371/journal.pmed.1002879 (PMC6685603; doi:10.1371/journal.pmed.1002879)
Supplement: S11 Appendix — (DOCX) [file pmed.1002879.s011.docx]

**Expectations of healthcare quality: a cross-sectional study of internet users in 12 low- and middle-income countries**

*S11 Appendix: Determinants of good ratings for poor quality, sub-analysis including self-reported mental health status*

|  | **Blood pressure visit; poor technical quality** | | | **Accident visit; poor technical quality** | | | **Blood pressure visit; poor interpersonal quality** | | | **Accident visit; poor interpersonal quality** | | |
| --- | --- | --- | --- | --- | --- | --- | --- | --- | --- | --- | --- | --- |
|  | **(n=17996)** | | | **(n=3640)** | | | **(n=3541)** | | | **(n=3667)** | | |
|  | Adjusted Odds Ratio | 95% CI | P-value | Adjusted Odds Ratio | 95% CI | P-value | Adjusted Odds Ratio | 95% CI | P-value | Adjusted Odds Ratio | 95% CI | P-value |
| **Individual Characteristics** | |  |  |  |  |  |  |  |  |  |  |  |
| Male gender | 1.28 | [1.18,1.39] | <.001 | 1.25 | [1.04,1.49] | 0.015 | 1.43 | [1.18,1.73] | <.001 | 1.19 | [1.01,1.41] | 0.035 |
| Age | 1.01 | [1.00,1.01] | 0.008 | 1.01 | [1.00,1.01] | <.001 | 1.01 | [1.00,1.01] | 0.129 | 1.00 | [1.00,1.01] | 0.268 |
| Educational attainment (ref: completed college or university) | | | | |  |  |  |  |  |  |  |  |
| -Some college/ university | 1.20 | [1.02,1.41] | 0.027 | 1.14 | [0.92,1.41] | 0.243 | 1.20 | [1.01,1.43] | 0.044 | 1.15 | [0.83,1.60] | 0.406 |
| -Secondary high school complete | 1.44 | [1.29,1.60] | <.001 | 1.53 | [1.25,1.88] | <.001 | 1.48 | [1.18,1.86] | 0.001 | 1.43 | [1.12,1.83] | 0.005 |
| -Some secondary or high school | 1.90 | [1.62,2.23] | <.001 | 1.90 | [1.45,2.49] | <.001 | 1.88 | [1.46,2.42] | <.001 | 2.17 | [1.49,3.17] | <.001 |
| -Primary school completed | 2.28 | [1.86,2.81] | <.001 | 2.34 | [1.76,3.10] | <.001 | 4.17 | [2.39,7.24] | <.001 | 1.90 | [1.16,3.09] | 0.010 |
| -Some primary school | 2.87 | [2.30,3.57] | <.001 | 4.01 | [2.59,6.22] | <.001 | 2.18 | [1.23,3.85] | 0.007 | 2.06 | [1.16,3.66] | 0.014 |
| -No formal schooling | 2.39 | [1.86,3.07] | <.001 | 1.82 | [1.15,2.87] | 0.010 | 2.39 | [1.75,3.27] | <.001 | 1.65 | [1.05,2.59] | 0.031 |
| Rural residence | 1.01 | [0.88,1.16] | 0.932 | 1.00 | [0.83,1.21] | 0.986 | 0.95 | [0.74,1.23] | 0.709 | 0.91 | [0.69,1.19] | 0.491 |
| Self-reported health (ref: poor) | | |  |  |  |  |  |  |  |  |  |  |
| -Fair | 0.88 | [0.73,1.05] | 0.155 | 1.03 | [0.68,1.55] | 0.896 | 0.95 | [0.57,1.58] | 0.838 | 0.89 | [0.60,1.30] | 0.538 |
| -Good | 1.46 | [1.11,1.92] | 0.006 | 2.14 | [1.34,3.41] | 0.001 | 1.49 | [0.87,2.56] | 0.145 | 1.36 | [0.88,2.10] | 0.168 |
| -Very good | 1.58 | [1.13,2.19] | 0.007 | 1.85 | [1.02,3.33] | 0.042 | 2.01 | [1.20,3.36] | 0.008 | 1.65 | [0.99,2.76] | 0.055 |
| -Excellent | 2.45 | [1.81,3.33] | <.001 | 3.22 | [1.72,6.04] | <.001 | 2.46 | [1.41,4.28] | 0.002 | 2.54 | [1.52,4.24] | <.001 |
| Self-reported mental health (ref: poor) | | |  |  |  |  |  |  |  |  |  |  |
| -Fair | 1.33 | [1.00,1.77] | 0.049 | 1.41 | [0.90,2.19] | 0.133 | 1.43 | [0.64,3.20] | 0.388 | 1.54 | [0.79,3.02] | 0.209 |
| -Good | 2.51 | [1.93,3.27] | <.001 | 2.00 | [1.36,2.94] | <.001 | 3.40 | [1.80,6.42] | <.001 | 2.49 | [1.46,4.24] | 0.001 |
| -Very good | 3.05 | [2.39,3.88] | <.001 | 2.79 | [1.74,4.46] | <.001 | 3.46 | [1.85,6.46] | <.001 | 3.21 | [1.83,5.64] | <.001 |
| -Excellent | 3.59 | [2.71,4.77] | <.001 | 3.00 | [1.70,5.28] | <.001 | 4.68 | [2.50,8.75] | <.001 | 4.60 | [2.54,8.30] | <.001 |
| Number outpatient visits in past year (ref: none) | 1.04 | [1.02,1.07] | <.001 | 1.06 | [1.04,1.08] | <.001 | 1.03 | [1.00,1.07] | 0.042 | 1.06 | [1.02,1.09] | 0.001 |
| Ever experienced discrimination (ref: no discrimination) | 1.51 | [1.33,1.71] | <.001 | 1.30 | [1.06,1.59] | 0.011 | 1.38 | [1.11,1.70] | 0.003 | 1.13 | [0.93,1.36] | 0.222 |
| **Country Characteristics** | | |  |  |  |  |  |  |  |  |  |  |
| Country variance | 0.10 | [.04, .22] |  | 0.05 | [.02,.15] |  | 0.21 | [.09,.50] |  | 0.21 | [.09,.49] |  |
| Number of countries | 12 | | | 12 | | | 12 | | | 12 | | |
| *Likelihood ratio test vs. logistic model* | *355.00* | | | *25.37* | | | *127.00* | | | *128.76* | | |
| *Prob. >= chibar2* | *<.001* | | | *<.001* | | | *<.001* | | | *<.001* | | |

*Caption: These results are from a mixed effects multilevel logistic regression. Coefficients are the adjusted odds ratio of having low expectations of care (defined as a rating of good or better on the vignettes). Data is unweighted. The prompt for number of visits was: “In the past year, how many times did you go to a clinic, health center, or hospital to receive health care for yourself? (Please do not include any times you stayed overnight.)”. The question regarding discrimination was: “Have you ever been discriminated against, hassled, or made to feel inferior by a health provider/staff for any of these reasons?” The likelihood ratio test compares this model to an ordinary logistic model.*
